# Biochanin A Induces S Phase Arrest and Apoptosis in Lung Cancer Cells

**DOI:** 10.1155/2018/3545376

**Published:** 2018-10-03

**Authors:** Yan Li, Haiyang Yu, Fengfeng Han, Mengmeng Wang, Yong Luo, Xuejun Guo

**Affiliations:** Department of Respiratory Medicine, Xin Hua Hospital Affiliated to Shanghai Jiao Tong University School of Medicine, China

## Abstract

Lung cancer is among the most common malignancies with a poor 5-year survival rate reaching only 16%. Thus, new effective treatment modalities and drugs are urgently needed for the treatment of this malignancy. In this study, we conducted the first investigation of the effects of Biochanin A on lung cancer and revealed the mechanisms underlying its potential anticancer effects. Biochanin A decreased cell viability in a time-dependent and dose-dependent manner and suppressed colony formation in A549 and 95D cells. In addition, Biochanin A induced S phase arrest and apoptosis and decreased mitochondrial membrane potential (ΔΨm) in A549 and 95D cells in a dose-dependent manner. Our results of subcutaneous xenograft models showed that the growth of Biochanin A group was significantly inhibited compared with that of control groups. Finally, P21, Caspase-3, and Bcl-2 were activated in Biochanin A-treated cells and Biochanin A-treated xenografts which also demonstrated that Biochanin A induced cell cycle arrest and apoptosis in lung cancer cells by regulating expression of cell cycle-related proteins and apoptosis-related proteins. In conclusion, this study suggests that Biochanin A inhibits the proliferation of lung cancer cells and induces cell cycle arrest and apoptosis mainly by regulating cell cycle-related protein expression and activating the Bcl-2 and Caspase-3 pathways, thus suggesting that Biochanin A may be a promising drug to treat lung cancer.

## 1. Introduction 

Lung cancer, also known as primary bronchogenic carcinoma, is one of the most common malignancies. Its incidence rate and fatality rates rank first among all types of cancers globally and tend to rise each year. Lung cancer has become the most serious of the malignant tumors and threatens the safety and the quality of human life worldwide. At present, research on the etiology, prevention, diagnosis, and treatment of lung cancer has become an important issue [1–3].

However, the limited progress in the treatment of lung cancer leads to a poor prognosis. The 5-year survival rate of lung cancer is only 16% since diagnosed. Therefore, research on the treatment of lung cancer and the development of new drugs are important for the prevention and cure of lung cancer. Chinese herbal medicine is an important part of Traditional Chinese Medicine. Research based on Chinese herbal medicine, especially based on a single ingredient, in the treatment of lung cancer is rare.

Therefore, the selection of a single ingredient from Chinese herbal medicine for treatment of tumors, especially for lung cancer, is a significant subject. Isoflavones are a type of plant estrogen in leguminous plants that have generally recognized uses in healthcare. Thus, Cicer arietinum L. isoflavones (i.e., Biochanin A) has attracted attention.

Pharmacological investigations have proven that Biochanin A can promote the excretion of terminal cholesterol products and help to reduce levels of blood sugar, blood fat, blood pressure, and liver tissue lipids. Cicer arietinum L. and its products are one of the best healthy for people with diabetes, high blood lipids, and hypertension. The existing literature shows that isoflavones also have anticancer pharmacological activity, especially for related cancers, such as breast cancer [4–8] and prostate cancer [9–13]. The mechanism of isoflavones' anticancer effects has not been fully elucidated.

Domestic and foreign research results have suggested several possible mechanisms, including one in which isoflavones induce cancer cell differentiation and apoptosis and which has a synergistic effect with cancer drugs.

Studies on the induction of apoptosis of cancer cells by isoflavones in vitro have shown that it interferes with the cell cycle of cells cultured in vitro, such as blocking the cell cycle of G1 / G2 phase of leukemia cells [14]. P21, the first CKI gene found, mainly acts by modulating the activity of CDK. P21 blocks the activity of all cyclin-CDK complexes, such as cyclin E-CDK2, cyclin D-CDK4, and cyclin A-CDK2. As a result, it has been suggested that P21 is associated with multiple processes in the cell cycle, and it is considered to be an important cancer gene. Studies in vitro have investigated whether isoflavones increase P21 expression in many types of cancer cells, such as breast cancer, prostate cancer, and small cell lung cancer. P21 also decreases the expression of cyclin B and blocks cells at the stage of G2/M. P21 may take part in an anticancer mechanism in which isoflavones induce cell apoptosis by activating cell apoptosis signaling. Tamura [15] has found that isoflavones, as plant estrogen, significantly decreased the Bcl-2/Bax ratio, thereby inducing the necrosis and apoptosis of tumor cells. Researchers have shown that TGF-*β* in liver cancer cells, gastric carcinoma cells, and breast cancer cells induces cell apoptosis by activating Caspase-3, inhibiting proteases, increasing Bax protein, and decreasing Bcl-2 protein and the HER-2 /neu gene [16].

In our early work, we have found that Biochanin A inhibits the proliferation of lung cancer cells. In the present study, the cell cycle analysis and flow cytometry results indicated that Biochanin A blocks lung cancer cells at the stage of G1/S and induces the apoptosis of lung cancer cells. However, the accurate molecular mechanism and the change of signal path in cell remain unknown.

Therefore, we hypothesized that Biochanin A inhibits cell proliferation by arresting the cell cycle at G1/S and promoting cell apoptosis. In the study, we investigated the cell viability, colony formation, mitochondrial membrane potential (ΔΨm), and expression level of cell cycle-related proteins (namely, cyclin A, P21, and CDK2) and apoptosis-related proteins (namely, Caspase-3, Bax, and Bcl-2) with the lung cancer cell line A549, 95D, and the subcutaneous xenograft models to explore the underlying mechanisms and molecular signaling pathways by which Biochanin A blocks the cell cycle and induces apoptosis.

## 2. Methods

A549 and 95D cells were purchased from the Shanghai Cell Institute Country Cell Bank. The cells were, respectively, cultured in high-glucose DMEM and RPMI-1640 medium (Gibco, USA) supplemented with 10% FBS (Gibco, USA) and 1% penicillin-streptomycin (Hyclone, USA). Both cell lines were cultured at 37°C and in 5% CO2.

To investigate the anticancer effect of Biochanin A in vitro, male nude mice ( 4–6 weeks old) purchased from the Shanghai Laboratory Animal Center of the Chinese Academy of Sciences (Shanghai, China) were used for the tumor xenograft models. All the experiments were approved by the Ethics Committee of Xinhua Hospital. Then tumor cells (A549 and 95D cells) were subcutaneously injected into the left axillae of the mice. The mice were divided into six groups (ten mice/group), of which half of the groups were injected with A549 cells and the other half were injected with 95D cells. One week later, the mice that were injected with A549 cells and the mice that were injected with 95D cells were exposed to three kinds of concentration gradient of Biochanin A i.p. (0, 15, and 60 mg/kg for the groups of A549 cells; 0, 18, and 72mg/kg for the groups of 95D cells) every three days. Tumor growth monitored every week was measured with the following formula: tumor volume (TV) = 4*π*/3 × (width/2)^2^ × (length/2). After 4 weeks, the mice were sacrificed, and the tumors were removed by dissection and weighed. Then the tumors were stored in the dark at −80°C for Western blotting.

### 2.1. Drugs and Antibodies

**Figure 1:**
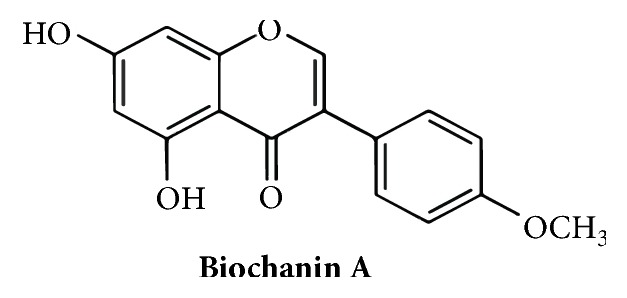
Chemical structure of Biochanin A. The molecular formula of Biochanin A is C16H12O5 and its molecular weight is 284.26.

Biochanin A was purchased from Jinhui Biology, dissolved in DMSO as a stock concentration of 100 mmol/L, and stored in the dark at −20°C.  Figure 1 shows the chemical structural of Biochanin A.

The different concentrations of Biochanin A used for different experiments were diluted with high-glucose DMEM. The antibodies used for Western blotting were as follows: rabbit anti-cyclin A, anti-P21, anti-Caspase-3, anti-Bcl-2, anti-Bax, and mouse anti-CDK2, anti-*β*-actin (Cell Signaling Technology, CST)

### 2.2. 3-(4, 5-Dimethylthiazol-2-yl)-2, 5-Diphenyltetrazolium (MTT) Assay

Drug sensitivity was detected with the MTT assay [17]. A549 and 95D cells were seeded into 96-well plates (Corning, USA) at a density of 5 × 10^3^ cells/well. After being cultured for 24 hours, the cells were then treated with fresh medium containing various concentrations (50, 100, 200, and 400 *μ*mol/L) of Biochanin A for 24, 48, and 72 h. Thereafter, we detected the cell viability with MTT assay at the absorbance of 490 nm on a microplate reader (Bio-Tek, USA). The experiments were repeated for three times and the cell viability was calculated according to the following formulation: cell viability = (OD treatment/OD control) × 100%.

### 2.3. Colony Formation Assay

In order to explore the effect of Biochanin A on colony formation, cells were seeded into 6-well plates (200 cells/mL). After adherence, we treated the cells with Biochanin A (0, 5, 10, and 20 *μ*mol/L) for 48 h and then cultured the cells for 15 days. Thereafter, the cells were fixed with 10% formalin and stained with crystal violet. We observed and took digital images of the stained single clones.

### 2.4. Cell Cycle Analysis

We performed Flow cytometric analysis to assess whether Biochanin A affected cell cycle progression in A549 and 95D cells. Being treated with different concentrations of Biochanin A (0, 50, 100, and 200 *μ*mol/L for A549 cells and 0, 60, 120, and 240 *μ*mol/L for 95D cells) for 48 h, the cells were collected and fixed with 70% ice-cold ethanol. Thereafter we stored the cells at -20°C. Then the cells were washed and resuspended in cold PBS. At last the cells were incubated at 37°C for 30 min with propidium iodide (Sigma-Aldrich). We used flow cytometry (BD, San Diego, USA) to analyze cell cycle distribution.

### 2.5. Flow Cytometric Analysis of Cell Apoptosis

We conducted the annexin V-FITC and propidium iodide staining (PI) to evaluate the effects of Biochanin A on apoptosis in A549 and 95D cells. Firstly, A549 and 95D cells were plated into 6-well plates (Corning, USA) and then incubated with Biochanin A (0, 50, 100, and 200 *μ*mol/L for A549 cells and 0, 60, 120, and 240 *μ*mol/L for 95D cells) for 48 h. The cells were collected, centrifuged, and then incubated with Annexin V- FITC/PI for 15 min at room temperature in the dark and was finally analyzed flow- cytometrically.

### 2.6. Detection of Mitochondrial Membrane Potential (ΔΨm) Variation with Fluorescence Microscopy (Rh 123 Staining)

To assess whether mitochondrial membrane integrity was damaged after treatment with Biochanin A, ΔΨm was detected by fluorescence microscopy using rhodamine 123, a yellow-green fluorescent probe. After treatment with Biochanin A (0, 50, 100, and 200 *μ*mol/L for A549 cells and 0, 60, 120, and 240 *μ*mol/L for 95D cells) for 48 h, we added 5 *μ*l of the JC-1 staining solution (Beyotime, China) per ml culture of medium to each well and then incubated samples for 20 min in darkness in a 5% CO2 incubator at 37°C. After being washed with buffer solution twice, A549 and 95D cells were analyzed by a fluorescence microscope (Leica, Germany).

### 2.7. Western Blot Analysis

A549 and 95D cells were treated with various concentrations of Biochanin A (0, 50, 100, and 200 *μ*mol/L for A549 and 0, 60, 120, and 240 *μ*mol/L for 95D) for 48 h; then A549 and 95D cells and the previous tumor tissues were lysed in lysis buffer (RIPA, 1 mM PMSF) and phosphatase inhibitors (Roche, Germany) to extract the total protein. Proteins were separated by SDS-PAGE (8%, 12% gels) under reducing conditions. The proteins were then electrophoretically transferred to nitrocellulose membranes. The membranes were blocked with 5% skimmed milk and incubated with anti-cyclin A, anti-P21, anti-Caspase-3, anti-Bcl-2, anti-Bax, anti-CDK2, and anti-*β*-actin antibodies, respectively (1:1000; Cell Signaling Technology) overnight at 4°C. This was followed by an incubation with goat anti-rabbit/anti-mouse secondary antibody (1:5000; Abcam). An equal loading of each lane was evaluated by immunoblotting the same membranes with anti-*β*-actin antibodies after the detachment of previous primary antibodies. The chemiluminescence reaction was detected by an ECL detection kit and the results were analyzed with the Gel Doc 2000 (Bio-Rad, USA).

### 2.8. Statistical Analysis

All values are expressed as the mean ±SD and were analyzed by Student's test using SPSS version 13.0 software. A value of less than 0.05 was considered statistically significant.

## 3. Results

### 3.1. Effects of Biochanin A on the Viability of A549 and 95D Cells

**Figure 2:**
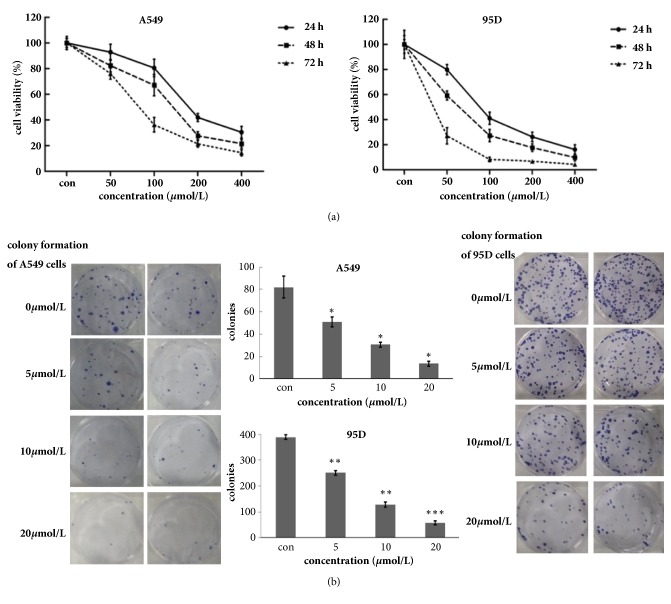
Biochanin A inhibits the proliferation of A549 and 95D cells. (a) Cells were treated with varying concentrations of Biochanin A, and the cell proliferation and IC 50 were determined by MTT assay on days 1, 2, and 3. Each value represents the mean ± SD (n = 3). (b) Biochanin A suppressed colony formation of A549 and 95D cells. Cells were treated with Biochanin A (0, 5, 10, and 20*μ*mol/L) and were allowed to form colonies in fresh medium for 14 days. These results were from 1 representative experiment of 3 independent trials. The photomicrographic difference (left panel) and influence of colonies (mean ± SD, n = 3) (right panel) in colony formation are shown. ^*∗*^p < 0.05; ^*∗∗*^p < 0.01; ^*∗∗∗*^p < 0.001.

The effects of Biochanin A on the growth of human A549 and 95D cells in vitro were tested. As analyzed by the MTT assay, after treatment for 24, 48, and 72 h, Biochanin A induced a dose- and time-dependent decrease in the viability of the A549 and 95D cells (Figure 2(a)). The ability of A549 and 95D cells to form colonies in the presence of Biochanin A was detected with the flat plate colony formation assay (Figure 2(b)). The colony count indicated that Biochanin A induced a dose-dependent decrease in colony formation ability of A549 and 95D cells. Moreover, control colony formation increased than those of the Biochanin A-treated group. The results suggest that Biochanin A may exert a significant influence on A549 and 95D cell proliferation.

### 3.2. Effects of Biochanin A on Cell Cycle Distribution

**Figure 3:**
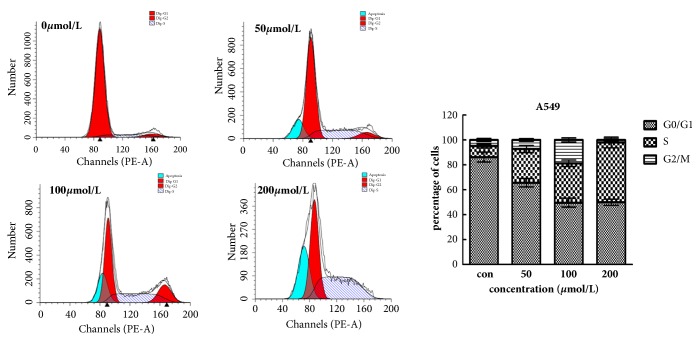
Biochanin A induces S phase arrest in A549. Cells were treated with 0, 50, 100, and 200 *μ*mol/L Biochanin A for 48 h and the DNA content was analyzed by flow cytometry. The percentages of cells in the G1, S, and G2/M phases of the cell cycle are shown. These results were from 1 representative experiment of 3 independent trials.

**Figure 4:**
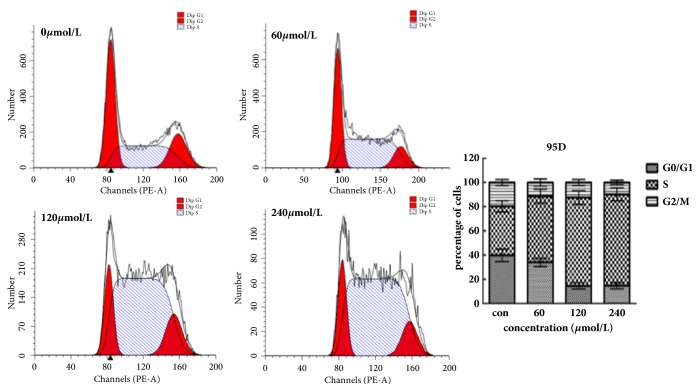
Biochanin A induces S phase arrest in 95D. Cells were treated with 0, 60, 120, and 240 *μ*mol/L Biochanin A for 48 h and the DNA content was analyzed by flow cytometry. The percentages of cells in the G1, S, and G2/M phases of the cell cycle are shown. These results were from 1 representative experiment of 3 independent trials.

After 48 hours of treatment with Biochanin A, the number of cells in the proliferative G0/G1 phase was significantly reduced, and the number of cells in the S phase was significantly increased. These results indicate that Biochanin A blocks the cell cycle in the S phase (Figures 3 and 4).

### 3.3. Effects of Biochanin A on Apoptosis in A549 and 95D Cells

**Figure 5:**
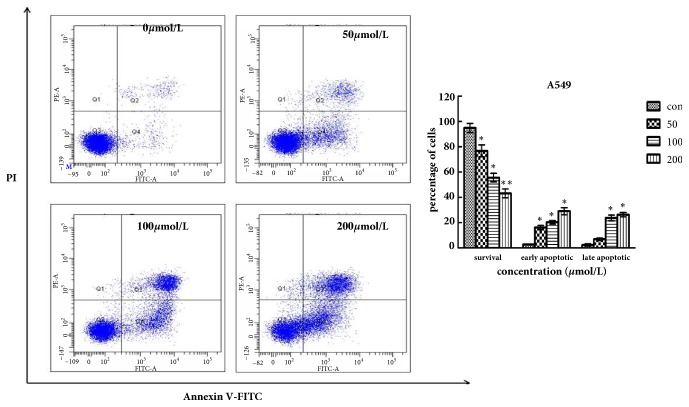
Biochanin A induces apoptosis in A549 cells. Cells were incubated with Biochanin A (0, 50, 100, and 200*μ*mol/L) for 48 h, followed by staining with annexin V/PI. The Q3 quadrant (annexin V−/PI−), Q4 quadrant (annexin V+/PI−), and Q2 quadrant (annexin V+/PI+) indicate the percentage of normal cells, early apoptosis, and late apoptosis, respectively. These results were from 1 representative experiment of 3 independent trials. Values represent the mean ± SD (n = 3). ^*∗*^*P* < 0.05; ^*∗∗*^*P*< 0.01; ^*∗∗∗*^*P* < 0.001.

**Figure 6:**
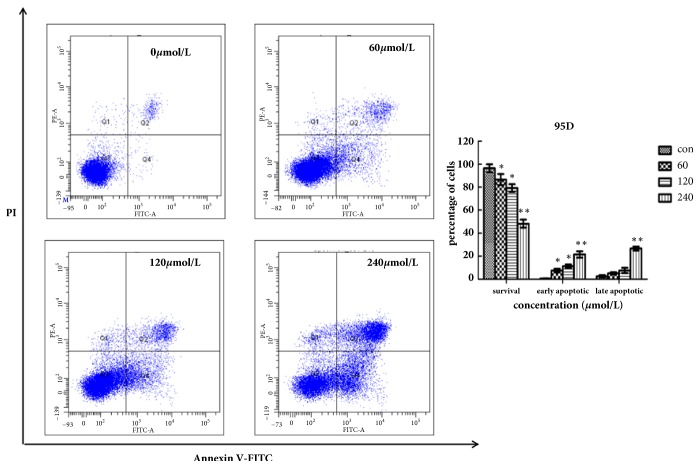
Biochanin A induces apoptosis in 95D cells. Cells were incubated with Biochanin A (0, 60, 120, and 240*μ*mol/L) for 48 h, followed by staining with annexin V/PI. These results were from 1 representative experiment of 3 independent trials. Values represent the mean ± SD (n = 3). ^*∗*^*P* < 0.05; ^*∗∗*^*P*< 0.01; ^*∗∗∗*^*P* < 0.001.

Flow cytometry results showed a significant dose-dependent increase in A549 and 95D apoptosis in both early and late stages of treatment with Biochanin A compared with the control group (Figures 5 and 6).

### 3.4. Biochanin A Decreases Mitochondrial Membrane Potential (ΔΨm)

**Figure 7:**
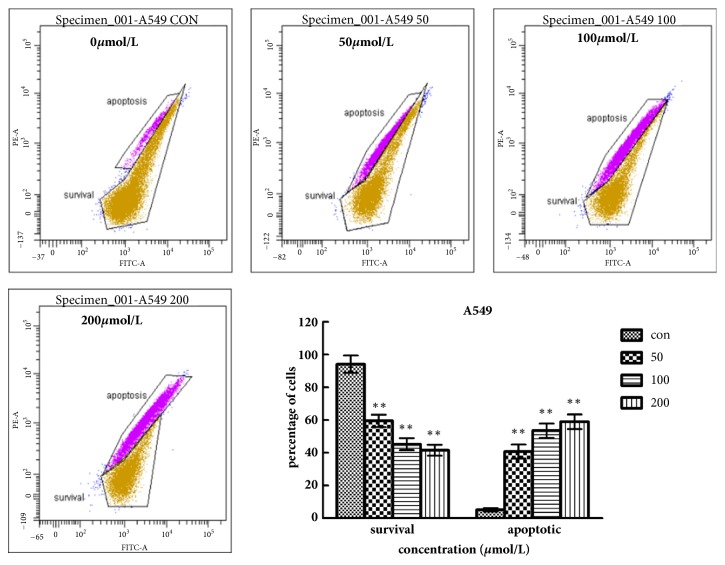
Biochanin A decreases mitochondrial membrane potential (ΔΨm) in A549 cells. Flow cytometric analysis of ΔΨm. A549 cells were treated with Biochanin A (0, 50, 100, and 200*μ*mol/L) followed by rhodamine 123 staining. Cells with high ΔΨm are marked “survival” and those with low ΔΨm are marked “apoptosis”. Percentages (%) of cells with high ΔΨm (survival) and low ΔΨm (apoptosis) are shown. These results were from 1 representative experiment of 3 independent trials. Values represent the mean ± SD (n = 3). ^*∗*^*P* < 0.05; ^*∗∗*^*P*< 0.01; ^*∗∗∗*^*P* < 0.001.

**Figure 8:**
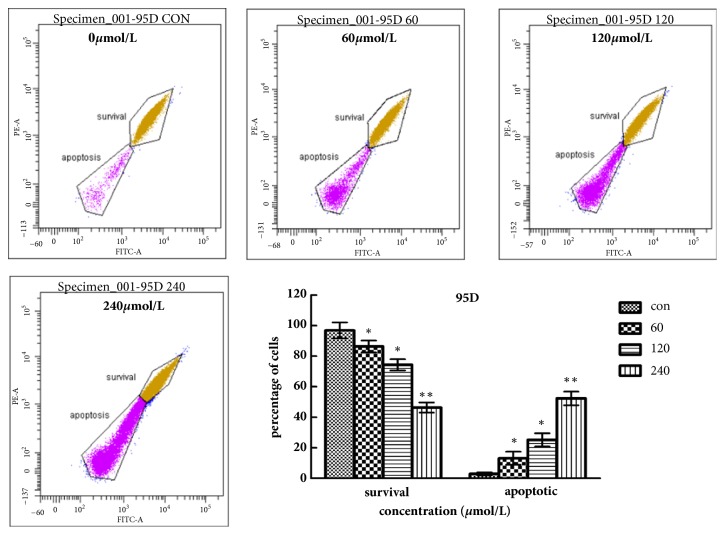
Biochanin A decreases mitochondrial membrane potential (ΔΨm) in 95D cells. Flow cytometric analysis of ΔΨm. 95D cells were treated with Biochanin A (0, 60, 120, and 240*μ*mol/L) followed by rhodamine 123 staining. Percentage (%) of cells with high ΔΨm (survival) and low ΔΨm (apoptosis) are shown. These results were from 1 representative experiment of 3 independent trials. Values represent the mean ± SD (n = 3). ^*∗*^*P* < 0.05; ^*∗∗*^*P*< 0.01; ^*∗∗∗*^*P* < 0.001.

As shown in Figures 7 and 8, Biochanin A induced a dose-dependent decrease in ΔΨm, which was manifested by an increase in the number of apoptotic cells with a low ΔΨm and a decrease in the number of viable cells with a normal ΔΨm. We found that most A549 cells and 95D cells showed a reduction in ΔΨm after treatment with 200 nmol/L and 240 nmol/L of Biochanin A for 48 h, respectively. These results indicated that a mitochondria-dependent mechanism was closely related to Biochanin A induced apoptosis in A549 and 95D cells.

### 3.5. Effects of Biochanin A on A549 and 95D Cell Growth In Vivo

**Figure 9:**
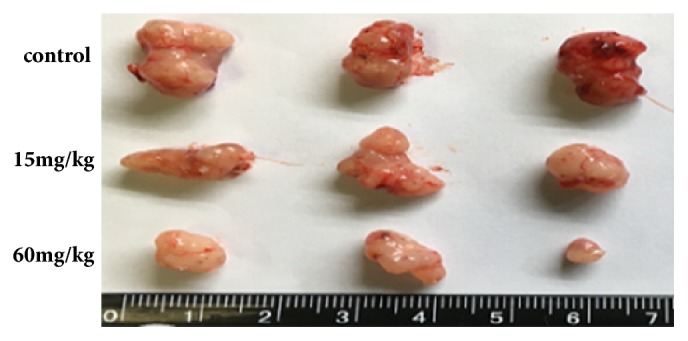
Biochanin A inhibits the proliferation of lung cancer cell lines A549 in vivo. Representative examples of tumors formed in nude mice injected with the indicated cells.

**Figure 10:**
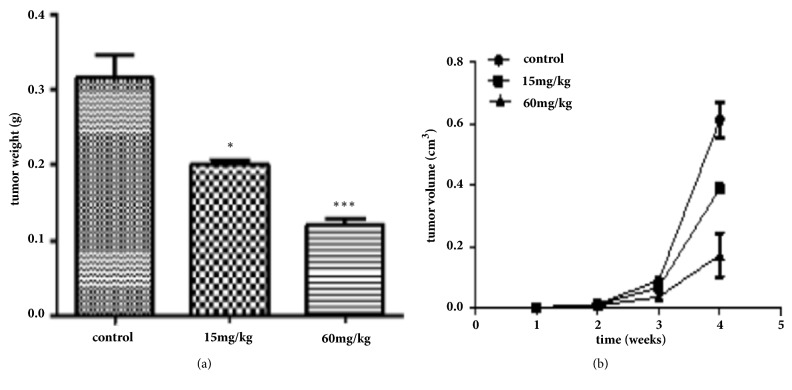
Biochanin A inhibits the proliferation of lung cancer cell lines A549 in vivo. (a) The bar graph of average tumor weights in the subcutaneous xenograft model. (b) Tumor growth curves are summarized in the line chart. ^*∗*^*P* < 0.05; ^*∗∗*^*P*< 0.01; ^*∗∗∗*^*P* < 0.001.

**Figure 11:**
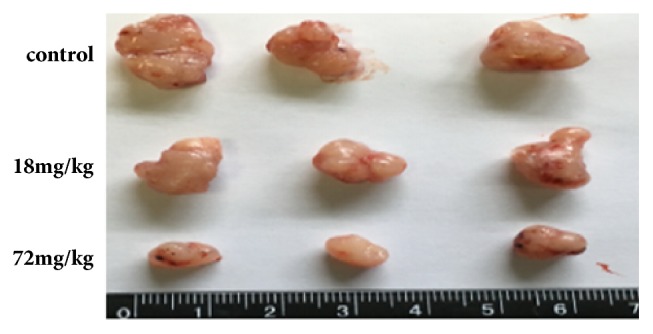
Biochanin A inhibits the proliferation of lung cancer cell lines 95D in vivo. Representative examples of tumors formed in nude mice injected with the indicated cells.

**Figure 12:**
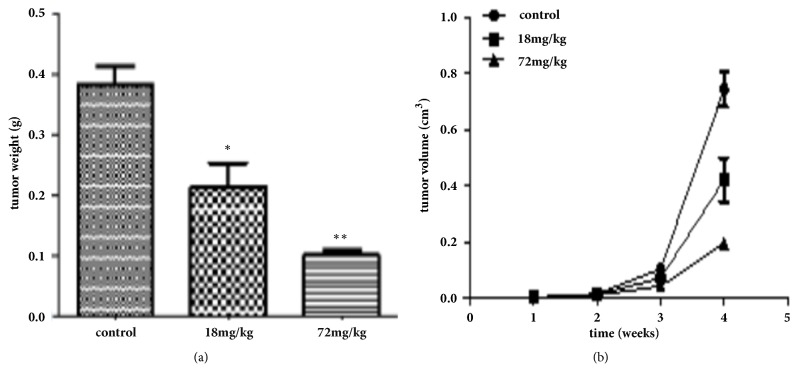
Biochanin A inhibits the proliferation of lung cancer cell lines 95D in vivo. (a) The bar graph of average tumor weights in the subcutaneous xenograft models. (b) Tumor growth curves are summarized in the line chart. ^*∗*^*P* < 0.05; ^*∗∗*^*P*< 0.01; ^*∗∗∗*^*P* < 0.001.

Furthermore, to assess the effects of A549 and 95D cell growth in vivo, A549 and 95D cells were injected into the left axillae of nude mice, and then different concentrations of Biochanin A were injected into different groups of nude mice. Finally, we investigated the tumor volume change and found that the growth of Biochanin A-treated xenografts was significantly inhibited compared with that of the control groups (Figures 9, 10, 11, and 12).

### 3.6. Effect of Biochanin A on the Signaling Pathway of Cell Cycle-Related Proteins and Caspase and Bcl-2 Family Members in A549 and 95D Cells

**Figure 13:**
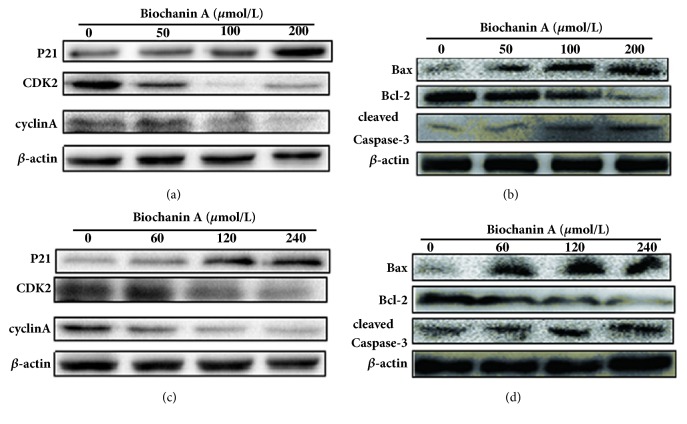
Effect of Biochanin A on the signal pathway of cell cycle-related proteins and apoptosis-related proteins in lung cancer cells in vitro. (a) The protein expression of cell cycle-related proteins in A549 cells in vitro. (b) The protein expression of apoptosis-related proteins in A549 cells in vitro. (c) The protein expression of cell cycle-related proteins in 95D cells in vitro. (d) The protein expression of apoptosis-related proteins in 95D cells in vitro.

**Figure 14:**
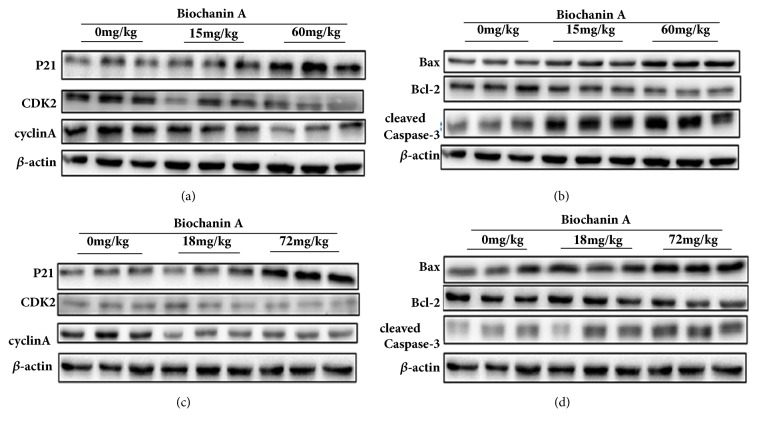
Effect of Biochanin A on the signal pathway of cell cycle-related proteins and apoptosis-related proteins in lung cancer cells in vivo. (a) The protein expression of cell cycle-related proteins in A549 cells in vivo. (b) The protein expression of apoptosis-related proteins in A549 cells in vivo. (c) The protein expression of cell cycle-related proteins in 95D cells in vivo. (d) The protein expression of apoptosis-related proteins in 95D cells in vivo.

To investigate the possible mechanism of Biochanin A-mediated cell cycle arrest and apoptotic effect on the lung cancer cells, the expressions of cell cycle-related proteins (namely, cyclin A, P21, and CDK2) and apoptosis-related proteins (namely, Caspase-3, Bax, and Bcl-2) were assessed by Western blot analysis. As illustrated in Figures 13(a), 13(b), 13(c), 13(d), 14(a), 14(b), 14(c), and 14(d), treatment with Biochanin A resulted in the downregulation of cyclin A, CDK2, and Bcl-2 and the upregulation of Bax, cleaved-Caspase-3, and P21, which may be partially responsible for the cell cycle arrest and apoptotic tendency of the A549 and 95D cells.

## 4. Discussion 

The existing literature has shown that isoflavones have anticancer pharmacological activity, and studies of isoflavone-mediated induction of cancer cell apoptosis in vitro have revealed that isoflavones have an interference effect on the cell cycle and can block the cell cycle at the G1/G2 stage in leukemia cells [14]. However, whether Biochanin A has anticancer pharmacological activity and its effect on lung cancer cells are unknown. In our study, we investigated the effect of Biochanin A on cell cycle arrest and apoptosis in lung cancer cells.

Our experiments showed that treatment with Biochanin A inhibited the viability and the growth of A549 and 95D cells in a time-dependent and dose-dependent manner. Two major regulatory mechanisms of cell growth are cell cycle regulation and apoptosis. When specific checkpoints during the cell cycle are arrested, apoptotic cell death occurs [18–23]. In our study, we observed that Biochanin A exhibits antitumor effects by inducing cell cycle arrest and apoptosis in vitro and in vivo, and we demonstrate the biochemical and molecular mechanisms that inhibit Biochanin A cell proliferation in lung cancer cells.

We performed Flow cytometric analysis to assess whether Biochanin A affected cell cycle progression in A549 and 95D cells. The results showed that after 48 hours of treatment with Biochanin A, the number of cells in the proliferative G0/G1 phase was significantly decreased, and the number of cells in the S phase was increased. This suggested that Biochanin A prevented DNA from replicating properly and blocked cells in S phase to inhibit tumor growth.

The CKI gene acts as a tumor suppressor that blocks the cyclin-CDK complex, while cyclins and cyclin-dependent kinases (CDKs) are two key classes of cell cycle regulatory molecules [24, 25]. Therefore, the CKI gene is thought to be involved in multiple processes of the cell cycle and acts as a brake to stop cell cycle progression [26]. P21 was the first CKI gene that was discovered. Overactivation of the P21 gene can result in cell cycle disorders and block cell proliferation [27]. Indeed, we observed a significant dose-dependent increase in the protein expression of P21 in both lung cancer cell lines and xenografts of lung cancer cell after treatment with Biochanin A. Furthermore, treatment with Biochanin A resulted in the downregulation of the protein expression of cyclin A and CDK2 in both lung cancer cell lines and xenografts of lung cancer cell. These results indicate that Biochanin A induces S phase arrest by regulating S phase-related protein expression in lung cancer cells.

We evaluated the effects of Biochanin A on apoptosis in lung cancer cell lines A549 and 95D by using annexin V-FITC and propidium iodide staining and Rh 123 staining. The results demonstrated a significant dose-dependent increase in the early and late stages of apoptosis in the Biochanin A-treated group compared to the control cells. As an autonomous cell death process, a variety of factors such as drugs and physical and chemical factors can induce apoptosis, which is considered to be an effective strategy for anticancer therapy [28, 29]. The family of cysteine-containing aspartate-specific proteases (caspase) contains many members that are associated with cell apoptosis [30–32]. Among the family of caspase, Caspase-3 is involved in the common pathway of cell apoptosis and is the key executor of cell apoptosis. Caspase-3 which is in the form of an inactive zymogen usually exists in the cytoplasm. When Caspase-3 is activated by many external apoptotic signals, many key proteases in the cytoplasm, nucleus, and cytoskeleton are inactivated, eventually leading to apoptosis. The results of our study showed that the expression of Caspase-3 was upregulated; this tendency coincided with the tendency of changes in cell apoptosis. The Bcl-2 gene family is antiapoptosis genes; its members are mainly divided into the proapoptotic protein Bax and antiapoptotic protein Bcl-2 according to their different biological effects [33, 34]. The Bcl-2/Bax ratio determined the occurrence and severity of apoptosis [35]. When The Bcl-2/Bax ratio decreased, it can induce the necrosis and apoptosis of tumor cells. The Western blot assay demonstrated that the expression of Bcl-2 was reduced, and the expression of Bax was increased in A549 and 95D cells treated with Biochanin A, thus significantly decreasing the Bcl-2/Bax ratio.

However, there are still some limitations about this study. In this study we implanted the lung cancer cells subcutaneously instead of orthotopically. Since the tumor can be produced in orthotopic implantation model with a more appropriate microenvironment, we can improve our research with orthotopic implantation model in the future study. Furthermore, a relatively high dose drug was applied in the study. We planned to explore effects at lower doses combined with traditional chemotherapeutic agents to better explore the drug effect.

## 5. Conclusion

In conclusion, these results of our study suggest that Biochanin A can inhibit the proliferation of lung cancer cells by inducing lung cancer cells cell cycle arrest and apoptosis mainly by regulating S phase-related protein expression and activating the Bcl-2 and Caspase-3 pathways.
